# Effect of acupuncture at Back-Shu points on gut microbiota in insomnia model rats based on metagenomic sequencing technology

**DOI:** 10.3389/fmicb.2025.1541958

**Published:** 2025-06-19

**Authors:** Shangwen Qi, Jiahui Qian, Yubing Li, Yujie Li, Wei Li, Xiyan Gao

**Affiliations:** ^1^Henan University of Chinese Medicine, Zhengzhou, Henan, China; ^2^The Third Affiliated Hospital of Henan University of Chinese Medicine, Zhengzhou, Henan, China; ^3^Institute of Acupuncture and Moxibustion, Henan University of Chinese Medicine, Zhengzhou, Henan, China

**Keywords:** metagenome, acupuncture, Back-Shu points, insomnia, gut microbiota

## Abstract

**Background:**

Increasing evidence indicates a bidirectional interaction between the gut microbiota and sleep regulation via the microbiota–gut–brain axis. Acupuncture is widely used to treat insomnia, and its efficacy may be mediated in part by modulation of the gut microbiota and its metabolic pathways.

**Methods:**

A rat model of insomnia was established by intraperitoneal injection of para-chlorophenylalanine (PCPA). Rats received acupuncture at Back-Shu points for 2 weeks. Sleep behavior was assessed using the pentobarbital-induced sleep test, and fecal samples were collected for metagenomic sequencing to analyze changes in gut microbial composition and function before and after acupuncture.

**Results:**

Compared with the model group, acupuncture significantly shortened sleep latency and prolonged sleep duration. Metagenomic analysis revealed that acupuncture partially restored the PCPA-induced decline in α-diversity and markedly altered β-diversity. Functionally, acupuncture enriched beneficial taxa such as Lactobacillus johnsonii and Ligilactobacillus murinus, and promoted pathways involved in tryptophan and glutamate metabolism as well as short-chain fatty acid (SCFA) synthesis. These changes may act by restoring neurotransmitter balance, strengthening gut barrier integrity, and modulating immune responses. Notably, SCFAs can activate G-protein–coupled receptors to suppress overactivation of the hypothalamic–pituitary–adrenal (HPA) axis, counteracting insomnia-related pathophysiology.

**Conclusion:**

Acupuncture at Back-Shu points ameliorates PCPA-induced insomnia-like behavior in rats and beneficially remodels gut microbiota structure and metabolic function. These findings support a key role for the microbiota–gut–brain axis in acupuncture’s regulation of sleep and provide a theoretical basis for developing microbiota-targeted adjunctive therapies for insomnia.

## 1 Introduction

Insomnia is primarily characterized by difficulty initiating sleep, problems maintaining sleep, and non-restorative sleep, and it is often accompanied by fatigue, depression, and anxiety (Perlis et al., 2022). As a chronic condition, insomnia has a persistent incidence rate ranging from 11 to 60% within 1 year (Ford and Kamerow, 1989; Morphy et al., 2007; Pillai et al., 2015; Pillai et al., 2016) and from 15 to 40% over 5 years of follow-up (Wong et al., 2016; Morin et al., 2020). Studies have shown that individuals with insomnia frequently experience intestinal microecological disturbances (Lin et al., 2024; Sejbuk et al., 2024), characterized by a reduction in beneficial bacteria and decreased microbial diversity (Lin et al., 2024). Dysbiosis of the gut microbiota can undermine mucosal barrier homeostasis, trigger low-grade systemic inflammation, and affect central nervous system function through the vagus nerve and immune pathways, potentially inducing or worsening insomnia symptoms (Pan et al., 2024).

Currently, treatment for insomnia largely relies on sedative medications such as benzodiazepines and antidepressants, which can provide rapid symptom relief. However, adverse effects such as tolerance, daytime drowsiness, and impaired cognitive function often make long-term adherence difficult (Madari et al., 2021; Sweetman et al., 2021). As a non-pharmacological intervention, acupuncture offers several advantages in treating insomnia, including reliable efficacy, few adverse reactions, and cost-effectiveness, and it has been widely applied in clinical practice (Zhao et al., 2024). Numerous clinical studies have confirmed that acupuncture can significantly improve sleep quality and prolong sleep duration, resulting in favorable therapeutic outcomes (Wang C. et al., 2021; Yeung et al., 2021; Lee et al., 2022).

Investigating the composition and genomic characteristics of the gut microbiota in patients with insomnia is essential for understanding the pathogenesis and mechanisms underlying the disorder. Nevertheless, there is still a lack of in-depth research on how acupuncture treatment may alter the gut microbiota’s species composition and genomic features in insomnia. Therefore, the present study aims to explore potential mechanisms by examining changes in the gut microbiota of an insomnia rat model before and after acupuncture treatment. This study was approved by the Ethics Committee of Henan University of Chinese Medicine (Approval No. IACUC-202302011).

## 2 Materials and methods

### 2.1 Experimental methods

#### 2.1.1 Animal grouping

Forty SPF-grade male SD rats (160–200 g, 6–8 weeks old) were provided by Jinan Pengyue Experimental Animal Breeding Co., Ltd. [License No. SCXK (Lu) 2022–0006]. They were housed at (22 ± 2)°C under a relative humidity of 60–70%, noise levels < 60 dB, and a 12/12 h light-dark cycle. All rats were certificated (Certificate No. 370726231100222618), ensuring the reliability and compliance of the experiments. After forty SD rats were acclimatized and fed for 7 days, they were randomized using SPSS25.0 software into the blank, model, AP, and estazolam groups, with 10 in each group. Except for the blank group, insomnia models were constructed by PCPA in each group. All experiment personnel were provided with pre-experimental training, and the basic conditions of the rats were recorded in detail every day during the experiment. The final test personnel were not involved in the animal grouping and did not participate in the recording, thereby ensuring the blinding of experimental observation and indicator analysis. The Animal Experiment Protocol has been reviewed and approved by Laboratory Animal Welfare and Ethics Committee of Henan University of Traditional Chinese Medicine [No. IACUC-202302011]. The study adhered to the ARRIVE guidelines.

#### 2.1.2 Insomnia modeling and evaluation

##### 2.1.2.1 Insomnia modeling

An aqueous solution of NaHCO3 at a concentration of 5% was prepared by gradually sprinkling sodium bicarbonate powder in pure water at 30–40°C. Then it was adjusted with 0.9% saline to be weakly alkaline (Ph 7–8). On this basis, the PCPA suspension (45 mg of PCPA per 1 mL) was prepared. To ensure the homogeneity of the suspension, the suspension was stirred by a magnetic stirrer for 10 consecutive hours. After preparation, the PCPA suspension was intraperitoneally injected into the rats at 1 mL/100 g every morning for three consecutive days in the model, AP, and estazolam groups. In the blank group, the rats underwent intraperitoneal injection of an equal amount of saline for three consecutive days during the same period, as controls.

##### 2.1.2.2 Model evaluation

To more accurately assess the establishment of the insomnia model, the pentobarbital sodium–induced sleeping test was performed. Twelve hours after the final injection of PCPA suspension, all rats were intraperitoneally administered pentobarbital sodium at 50 mg/kg. Their behavioral responses were closely observed. The disappearance of the righting reflex for 1 min was considered the sleep onset latency. The righting reflex—an instinctive response by which rats regain normal posture after losing balance—serves as a critical indicator of falling asleep. Recovery of the righting reflex was defined as at least two righting movements within 60 s, signifying the end of sleep; this duration was recorded as the sleep duration. Sleep latency and duration were meticulously documented for each rat, and one-way ANOVA was used to assess the effects of PCPA on sleep. Significantly prolonged sleep latency and shortened sleep duration in the model group, with statistical difference from the blank group, were interpreted as indicative of successful insomnia modeling (Tian et al., 2024).Moreover, to minimize potential interference with subsequent metagenomic sequencing, fecal samples were collected 24 h after the completion of all sleep experiments and the final acupuncture and gavage procedures. This ensured that the rats had returned to their natural physiological state, reducing the influence of anesthetics on the gut microbiota and enhancing the reliability and stability of the sequencing results.

### 2.2 Operation in each group

#### 2.2.1 AP group

The rats were treated with bilateral AP at Back-Shu points of the five Zang-organs (Xinshu, Ganshu, Pishu, Feishu, and Shenshu), and Experimental Acupuncture (Xu et al., 2019) was consulted for the rat AP site

Specifically, fixed personnel were responsible for the operation of AP, achieving standardization and consistency of the whole process. Before AP, the local skin of the rats was thoroughly sterilized to ensure the cleanliness and sterility of the operation. The rat was placed in a self-made fastening bag and immobilized on an operating table in a prone position, thereby maintaining postural stability and reducing the error caused by movement during AP. Meanwhile, the surface of the fixing band was also sterilized to further ensure the hygiene and safety of the operation. A disposable AP needle (0.25 × 15 mm) was straight inserted to a depth of 5 mm, without adopting special techniques, and it was retained for 15 min to stimulate the points and exert a therapeutic effect.

In addition, AP was performed at 9:00 a.m. daily only once, thereby ensuring the consistency and comparability of the experiments. In this way, all rats were in the same state and environmental conditions when undergoing AP. AP lasted for seven consecutive days to fully observe the impact and effect of AP on the rats.

#### 2.2.2 Estazolam group

The rats were given Estazolam Tablets (Huazhong Pharmaceutical Co., Ltd., Item No.: H42021522), a commonly used drug manufactured by Huazhong Pharmaceutical Co., Ltd. Specifically, Estazolam Tablets were ground into fine powder, mixed with purified water at 0.08 mg/mL, and stirred thoroughly until it was dissolved evenly, which was essential for ensuring that each rat was given an accurate and consistent dose. The drug was administered at 0.558 mg/kg by gavage using a 12-gauge curved gavage needle, so that it could be sent to the stomach smoothly. The gavage was performed at 9:00 a.m. daily to ensure consistency of experimental conditions. The treatment lasted for seven consecutive days to observe the long-term effect of estazolam and compare it with other groups.

The principles of ethics in animal experimentation were strictly observed throughout the experiment to ensure sterile or clean operations and minimize discomfort and pain to the rats. Meanwhile, the responses and status of the rats were closely observed during the experiment, and the experimental conditions were promptly adjusted to ensure accuracy and reliability.

#### 2.2.3 Blank group

The rats were caught and immobilized in the same way as the AP and estazolam groups.

#### 2.2.4 Model group

The rats were caught and immobilized in the same way as the AP and estazolam groups.

#### 2.3 Sampling

Samples were taken 24 h after the end of the last treatment to exclude the potential influence of food on the results of subsequent experiments, thereby guaranteeing the result accuracy and satisfying the basic physiological needs of the rats. Specifically, all rats were deprived of food for 24 h but were given water. Then they were injected intraperitoneally with 1.5% pentobarbital sodium at 150 mg/kg (the required dose of pentobarbital sodium was accurately calculated based on the body weight measured before the injection). The anesthetized rats were placed supine on an operating table, and skin preparation was first performed on the abdomen using electric hair cutters for further processing of the colon tissues. The colon tissues were cut at 2 cm distal to the cecum, and then the colon content was harvested to collect the metabolites for later metagenome analyses.

### 2.4 Indicator detection

#### 2.4.1 Procedures for metagenome detection

(1) Sample extraction and detection: From the rat colon content, genomic DNA was extracted utilizing the CTAB method, and its concentration, integrity, and purity were detected by Agilent5400.
(2) Library preparation and retrieval: A library was prepared using NEB Next^®^ Ultra DNA Library Prep Kit for Illumina (NEB, United States). After utilizing the Covaris ultrasonic crusher to randomly cleave qualified DNA samples into fragments of around 350 bp, the DNA fragments were end-repaired, poly-A-tailed, and ligated with the adapter, followed by purification and PCR amplification. Finally, the AMPure XP system was used to purify the PCR products, the Agilent2100 was used to quantify the library’s insert size, and real-time PCR was conducted to determine the library concentration.
(3) Sequencing: The Illumina PE Cluster Kit (Illumina, United States) was used to cluster the index-coded samples using a cBot Cluster Generation System in accordance with the manufacturer’s instructions. Following cluster generation, paired-end 150 bp reads were produced by sequencing the library on an Illumina NovaSeq 6000.
(4) Data quality control and de-hosting: To acquire raw metagenome data (Raw Data) of bacteria, fungi, and viruses in rat colon content samples, metagenome sequencing was carried out on Illumina NovaSeq. We preprocessed the raw sequencing data (Raw Data) using KneadData in the following ways to guarantee data reliability: (1) The raw data were cleansed of adapter sequences (Trimmomatic, ILLUMINACLIP:adapters_path:2:30:10), sequences with a final length less than 50 bp (Trimmomatic, MINLEN:50), and low-quality (default threshold ≤ 20) sequences (Trimmomatic, SLIDINGWINDOW:4:20). (2) In light of the potential for host contamination in the samples, Clean Data should be aligned to the host genome, and Bowtie2 (Parameter: –very-sensitive) was used by default to filter the valid sequences from the host for later analyses. (3) Finally, FastQC was adopted for the rationality and effectiveness of quality control (Martin, 2011; Schmieder and Edwards, 2011; Langmead and Salzberg, 2012; Kechin et al., 2017).
(4) Species annotation: By aligning the samples with Kraken2 and the microbial nucleic acid database (bacterial, fungal, archaeal, and viral sequences were screened from the NCBI NT and the RefSeq), the number of sequences of the species was determined. Bracken then forecasted the actual relative abundance of the species. Kraken2 is the most recent K-mer-based alignment program. 16,799 known bacterial genomes were found in local Kraken2 (Wood and Salzberg, 2014; Brum et al., 2015; Mandal et al., 2015; Lu et al., 2017).
(5) Reads-based functional annotation: After the quality controlled and de-host sequences were aligned with the protein database (UniRef90) using HUMAnN2 software, a relative abundance table and annotation data for each functional database were obtained from the correspondence between the UniRef90 ID and database (Zhu et al., 2010; Segata et al., 2011; Kim et al., 2016; Franzosa et al., 2018). The species and functional abundance tables were used to perform sample clustering analysis, abundance clustering analysis, PCoA, and NMDS dimensionality reduction analysis (species only). The Dunn test and LEfSe biomarker analysis were then conducted using the grouping data to evaluate differences in species and functional composition (Villar et al., 2015).
(6) DIAMOND was used to align the quality-controlled and de-host sequences of the samples with the CARD database, and the sequences that failed to be aligned [parameter: -e0.001 (e-value < 1e-3)-i80 (percent identity > 80%)] were filtered out. The relative abundance of antibiotic-resistant genes in samples was determined based on the alignment findings (Buchfink et al., 2015).

### 2.5 Statistical processing

Metagenome data were analyzed using species accumulation curves, which were effective in measuring and predicting how species abundance within microbiota increases with an increasing sample size. R4.2.1 was utilized to calculate the alpha diversity of species (Chao1, Shannon, and Simpson), which provided a multidimensional view of species diversity. The Wilcoxon rank sum test was conducted for comparison, and P < 0.05 indicated statistically significant. In addition, significant differences in microbiota were revealed by beta diversity analyses, and the significance level was also set to *P* < 0.05. To dig deeper into the differences in biomarkers among groups, statistically significant biomarkers were searched for among groups by LEfSe analysis, thereby supporting the subsequent biological interpretation. At the functional level, the Reporter Score was applied to statistically test all KOs (KEGG Orthology, i.e., clusters of functionally similar genes) involved in specific pathways. When the absolute value of the enrichment score, i.e., Reporter Score, exceeded the predefined threshold, the pathway enrichment was considered significantly different. Finally, bar graphs were drawn based on the information of pathways in the KEGG to visualize the variation in the relative abundance of microbiota. These graphs not only revealed the enrichment of specific pathways among groups but also provided a visual perspective for understanding the functional differences of microbiota. *P* < 0.05 was set as the significance level and was deemed statistically significant rather than resulting from random errors. In this way, we could more accurately identify differences among groups and obtain strong statistical support for subsequent studies.

## 3 Results

### 3.1 Changes in sleep duration after modeling

The results showed significant differences in sleep latency and sleep duration between the control group and the three PCPA-treated groups. Following PCPA administration, sleep latency in the three model groups was markedly prolonged and sleep duration significantly shortened compared with the control group (*P* < 0.01), indicating successful establishment of the PCPA-induced insomnia model. The observed prolongation of sleep latency and reduction in sleep duration in the model groups were consistent with the expected effects of PCPA (Table 1 and Supplementary Figure S1).

**TABLE 1:** Changes in sleep duration after modeling ($\bar{\chi }$ ± s).

Group	*n*	Sleep latency (min)	Sleep duration (min)
Blank group	10	10.97 ± 1.91	88.43 ± 3.97
Model group	10	20.88 ± 2.89***	55.91 ± 5.03***
AP group	10	19.41 ± 2.61***	54.18 ± 6.38***
Estazolam group	10	20.60 ± 2.92***	56.17 ± 5.96***

Data are expressed as mean ± standard deviation. *P < 0.05, **P < 0.01,

***P < 0.001, compared with the blank group.

### 3.2 Changes in sleep duration after treatment

After treatment, rats in the acupuncture group exhibited a significantly shorter sleep latency and a markedly longer sleep duration compared with the model group (*P* < 0.001). Similarly, the estazolam group showed a significant reduction in sleep latency and an increase in sleep duration versus the model group (*P* < 0.01). Although differences between the acupuncture and estazolam groups did not reach statistical significance (*P* > 0.05), the acupuncture group demonstrated a more pronounced trend toward improved sleep latency and prolonged sleep duration, suggesting a potential advantage of acupuncture.

Moreover, as a non-pharmacological therapy grounded in traditional Chinese medicine, acupuncture modulates organ function to normalize sleep without disrupting metabolic processes. This holistic mechanism underscores its unique and systematic benefits in insomnia intervention, indicating strong clinical applicability and translational value (Table 2 and Supplementary Figure S2).

**TABLE 2:** Changes in sleep duration after treatment ($\bar{\chi }$ ± s).

Group	*n*	Sleep latency (min)	Sleep duration (min)
Blank group	10	10.34 ± 1.4	85.41 ± 3.1
Model group	10	25.27 ± 2.28***	44.43 ± 5.95***
AP group	10	17.78 ± 2.27***	71.77 ± 3.56***
Estazolam group	10	14.63 ± 1.63***	76.83 ± 4.54**

Data are expressed as mean ± standard deviation. *P < 0.05,

**P < 0.01,

***P < 0.001, compared with the blank group.

### 3.3 Metagenome analyses

#### 3.3.1 Generation of basic sequencing data

Some low-quality data were present in the Raw Data obtained by Illumina sequencing. Preprocessing including quality control and de-hosting was required for the Raw Data to acquire valid sequences for subsequent analyses, thereby ensuring the accuracy and reliability of subsequent analysis results. The statistical results of sequencing data preprocessing are shown in the table below. The critical parameters in the quality control steps are interpreted as follows (Table 3).

**TABLE 3:** Sequencing data preprocessing statistics.

Sample	Raw reads	Raw base (GB)	Raw Q20 (%)	Raw Q30 (%)	Clean reads	Cleaned	Clean Q20 (%)	Clean Q30 (%)
V1	22,346,168	6.7	97.4	92.91	15126579	67.69	98.61	94.86
V2	20,231,258	6.07	97.43	92.93	19344848	95.62	98.54	94.7
V3	22,674,174	6.8	97.4	92.83	21260640	93.77	98.52	94.62
V4	22,163,987	6.65	97.43	92.87	21100169	95.2	98.53	94.63
V5	22,153,733	6.65	97.38	92.79	21007154	94.82	98.51	94.57
V6	21,592,548	6.48	97.56	93.17	20604839	95.43	98.6	94.83
V7	2,0921,963	6.28	97.53	93.11	20070562	95.93	98.57	94.76
V8	21,074,427	6.32	97.47	92.98	19721477	93.58	98.6	94.81
V9	20,580,885	6.17	97.47	93.01	19565238	95.07	98.58	94.79
V10	20,055,088	6.02	97.55	93.2	18867944	94.08	98.63	94.94
W1	20,951,539	6.29	97.45	92.92	20071560	95.8	98.53	94.62
W2	22,685,100	6.81	97.51	93.07	21710482	95.7	98.58	94.79
W3	23,185,933	6.96	97.48	92.96	22231712	95.88	98.53	94.64
W4	22,167,079	6.65	97.54	93.08	20658955	93.2	98.62	94.83
W5	20,449,090	6.13	97.54	93.14	19430227	95.02	98.63	94.89
W6	21,779,824	6.53	97.4	92.77	18516633	85.02	98.53	94.58
W7	21,303,280	6.39	97.49	93.04	19331803	90.75	98.59	94.8
W8	20,623,156	6.19	97.45	93	19693642	95.49	98.56	94.76
W9	22,823,013	6.85	97.51	93.04	21137494	92.61	98.56	94.72
W10	20,895,842	6.27	97.56	93.19	19541689	93.52	98.61	94.87
X1	22,825,567	6.85	97.54	93.11	21850869	95.73	98.58	94.76
X2	22,910,883	6.87	97.48	92.98	21799928	95.15	98.57	94.73
X3	20,063,158	6.02	97.44	92.89	18230750	90.87	98.55	94.67
X4	19,710,018	5.91	97.32	92.73	12523527	63.54	98.59	94.79
X5	20,771,839	6.23	97.46	92.95	18949503	91.23	98.57	94.72
X6	22,823,105	6.85	97.51	93.06	21645352	94.84	98.57	94.75
X7	202,192,51	6.07	97.46	93.01	19148081	94.7	98.57	94.77
X8	21,664,371	6.5	97.4	92.86	20714344	95.61	98.52	94.63
X9	22,360,208	6.71	97.41	92.72	21392025	95.67	98.45	94.36
X10	21,931,741	6.58	97.42	92.82	19844430	90.48	98.52	94.55
Y1	21,272,260	6.38	97.52	93.08	20154216	94.74	98.58	94.77
Y2	22,420,244	6.73	97.37	92.78	21437966	95.62	98.49	94.54
Y3	22,897,120	6.87	97.4	92.82	21492380	93.86	98.52	94.6
Y4	22,746,620	6.82	97.37	92.48	21186659	93.14	98.37	94.07
Y5	22,704,180	6.81	97.31	92.62	21688986	95.53	98.45	94.41
Y6	22,391,176	6.72	97.5	92.97	20038621	89.49	98.54	94.63
Y7	23,007,150	6.9	97.3	92.66	21493947	93.42	98.48	94.52
Y8	19,829,944	5.95	97.47	92.99	18525995	93.42	98.58	94.78
Y9	20,695,754	6.21	97.45	92.99	16690226	80.65	98.62	94.88
Y10	22,145,526	6.64	97.37	92.83	14651280	66.16	98.57	94.74

Sample, sample name; Raw reads, the number of Raw sequencing reads; Raw Base (GB), the number of Raw reads in GB, and the total number of bases in the raw sequencing data was calculated by the number of Raw reads × the sequencing length; Clean Reads, the number of Clean reads after filtering (quality control and de-hosting); Cleaned, the percentage of residual sequences in Raw reads after filtering; Q20, the percentage of bases with quality scores > 20; Q30, the percentage of bases with quality scores > 30; V, estazolam group; W, blank group; X, model group; Y, AP group.

(1) Removal of adapter sequences (ILLUMINACLIP: adapters_path:2:30:10).
(2) Removal of subsequent sequences (SLIDINGWINDOW: 4:20) if the average quality score was below 20 (99% accuracy) after sequence scan (sliding window of 4 bp).
(3) Removal of sequences with a final length less than 50 bp (MINLEN:50).

#### 3.3.2 Analysis of species composition

**FIGURE 1:**
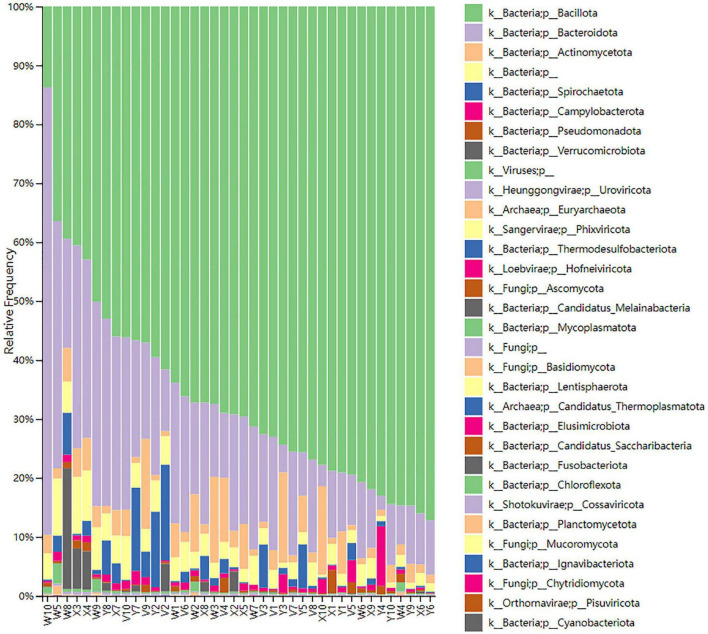
Relative abundance of gut microbiota at the phylum level across different groups. The dominant phyla in all groups included Bacillota, Bacteroidota, and Actinomycetota, followed by Spirochaetota, Campylobacterota, and Pseudomonadota. The microbial composition of the blank group (W) differed markedly from the other groups, suggesting regulatory effects of different interventions on the gut microbiota. W, blank group; X, model group; Y, acupuncture (AP) group; V, estazolam group.

**FIGURE 2:**
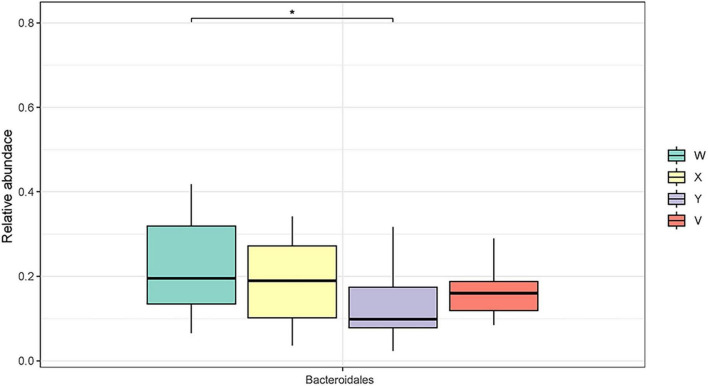
Relative abundance of the order Bacteroidales in fecal samples of each group. The abundance of Bacteroidales was significantly higher in the blank group than in the model and intervention groups (*P* < 0.05), indicating it may be a characteristic taxon associated with normal sleep states. W, blank group; X, model group; Y, AP group; V, estazolam group. The asterisk “*” denotes statistical significance at *p* < 0.05 (versus the comparison group specified in each panel).

**FIGURE 3:**
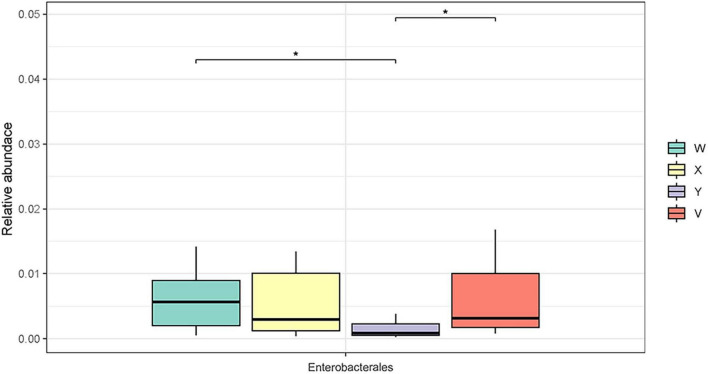
Relative abundance of the order Enterobacterales in each group. Enterobacterales abundance was elevated in the model group but significantly reduced in the acupuncture group (*P* < 0.05), potentially reflecting the inhibitory effect of acupuncture on harmful bacteria. The asterisk “*” denotes statistical significance at *p* < 0.05 (versus the comparison group specified in each panel).

The relationship between gut microbiota composition and treatment response was assessed by comparing changes in microbial relative abundance and diversity across the different rat groups. At the phylum level, Bacillota, Bacteroidota, and Actinomycetota were the dominant communities, followed by Spirochaetota, Campylobacterota, and Pseudomonadota (Figure 1). At the order level, a similar distribution pattern was observed. For example, Bacteroidales (phylum Bacteroidota) exhibited the highest relative abundance in the control group, significantly exceeding that of the model group (*P* < 0.05; Figure 2). Conversely, Enterobacterales (phylum Proteobacteria) was significantly reduced in the acupuncture group compared both to the model group and to the estazolam group (*P* < 0.05; Figure 3), suggesting that acupuncture may suppress certain pathogenic Gram-negative bacteria.

**FIGURE 4:**
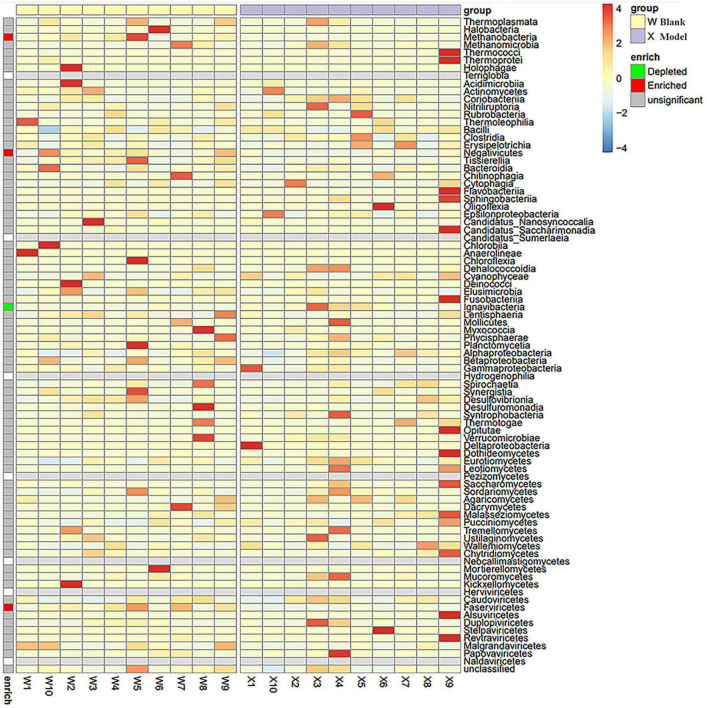
LEfSe analysis (phylum level) of significantly different taxa between the blank and model groups. Compared with the model group (X), the blank group (W) showed significant enrichment of Methanobacteria (*P* < 0.01), Faserviricetes (*P* < 0.01), and Negativicutes (*P* = 0.035), while Ignavibacteria (*P* = 0.024) was elevated in the model group, suggesting their potential roles in the development and recovery of insomnia. W, blank group; X, model group.

**FIGURE 5:**
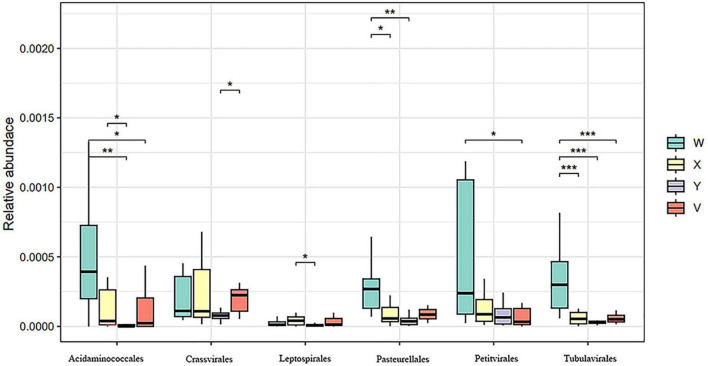
Relative abundance and statistical comparison of microbial taxa at the order level among groups. Acidaminococcales and Pasteurellales were dominant in the blank group, while Leptospirales had relatively low abundance. The asterisks indicate levels of statistical significance: *P < 0.05; **P < 0.01; ***P < 0.001.

Most taxa showing statistically significant differences belonged to Bacillota and Bacteroidota. LEfSe analysis (Figure 4) further revealed that Methanobacteria (*P* < 0.01), Faserviricetes (*P* < 0.01), and Negativicutes (*P* = 0.035) were enriched in the control group relative to the model group, whereas Ignavibacteria (*P* = 0.024) was enriched in the model group, indicating these characteristic taxa may be closely linked to insomnia onset and recovery. As shown in Figure 5, Acidaminococcales and Pasteurellales dominated in the control group—potentially reflecting associations with normal sleep—while Leptospirales remained at low abundance. Additionally, Tubulavirales differed significantly among the control, model, acupuncture, and estazolam groups (*P* < 0.001), pointing to its possible role in insomnia pathophysiology or therapeutic intervention.

**FIGURE 6:**
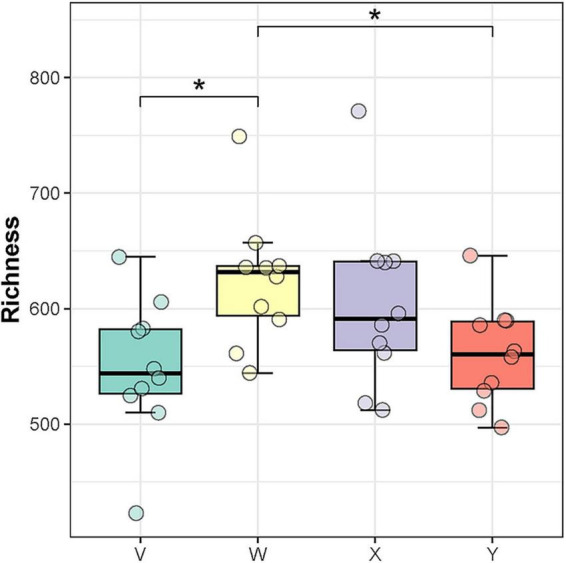
Comparison of gut microbiota richness among groups. Microbial richness was significantly increased in the model group, indicating microbial dysbiosis. The estazolam and acupuncture groups showed diversity levels closer to the blank group, suggesting partial restoration of gut microbial homeostasis after intervention (*P* < 0.05). The asterisk “*” denotes statistical significance at *p* < 0.05 (versus the comparison group specified in each panel).

**FIGURE 7:**
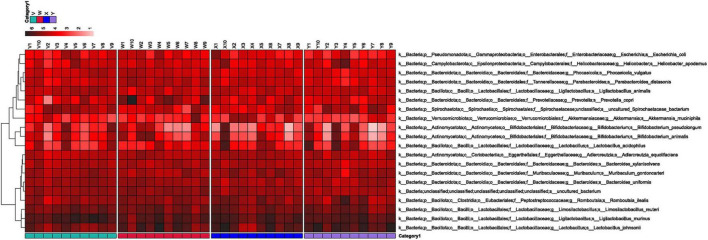
Hierarchical clustering heatmap based on the top 20 most abundant species. Samples from different groups exhibited distinct clustering patterns. The microbial composition in the acupuncture group tended to shift toward that of the blank group, indicating a favorable effect of acupuncture on microbiota modulation. W, blank group; X, model group; Y, acupuncture group; V, estazolam group.

Figure 6 illustrates that microbial richness was significantly elevated in the model group, indicating dysbiosis. Post-intervention, the estazolam group’s microbiota trended toward stability, and the acupuncture group’s community shifted closer to that of the control group; notably, the two treatment groups exhibited high similarity, suggesting a favorable microecological regulatory effect of acupuncture. Hierarchical clustering of the top 20 most abundant taxa (Figure 7) further revealed distinct grouping patterns across the four groups, underscoring differential impacts of each intervention on the gut microbiome. In particular, the acupuncture group’s profile more closely resembled the normal physiological state, reflecting the holistic regulatory principles of traditional Chinese medicine—namely, “balance of yin and yang” and “unblocking the flow of qi.”

In summary, gut microbiome alterations were closely associated with both insomnia modeling and its treatment. By modulating specific microbial communities, acupuncture may influence host sleep rhythms, metabolism, and neuroimmune status, supporting the potential of gut microbiota as a biomarker for acupuncture-based insomnia therapy.

#### 3.3.3 Significance analysis of species differences among groups

Various microecological analyses were conducted to evaluate the regulatory effects of acupuncture and estazolam on the gut microbiota of insomnia model rats.

**FIGURE 8:**
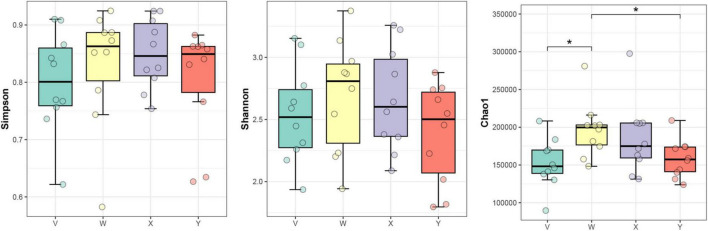
Comparison of α-diversity indices (Chao1, Shannon, and Simpson) among groups. Chao1 index differed significantly among the blank, acupuncture, and estazolam groups (*P* < 0.05), suggesting differential impacts of interventions on species richness. Shannon and Simpson indices showed no significant changes, indicating community evenness remained stable. Box plots were analyzed using one-way ANOVA. The asterisk “*” denotes statistical significance at *p* < 0.05 (versus the comparison group specified in each panel).

α-Diversity. Species richness and evenness were assessed using the Chao1, Shannon, and Simpson indices. The Shannon and Simpson indices did not differ significantly among groups, indicating that overall community diversity remained stable. However, the Chao1 index was significantly higher in the acupuncture group compared with both the control and estazolam groups (*P* < 0.05), suggesting that acupuncture specifically modulates species richness (Figure 8).

**FIGURE 9:**
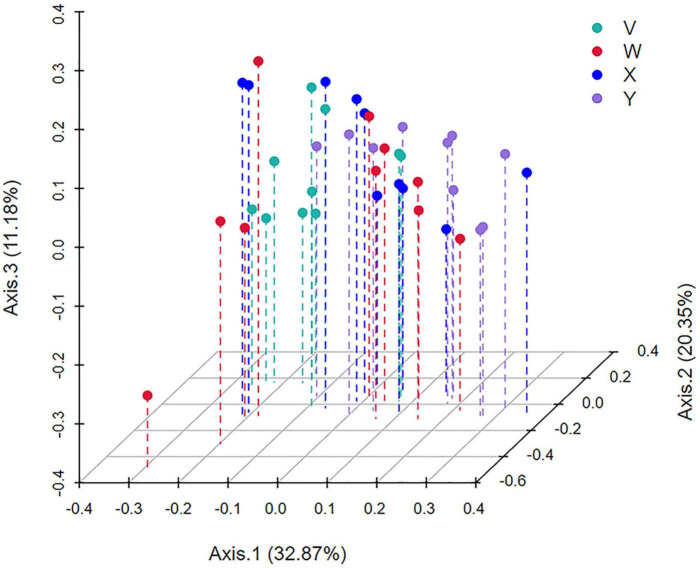
Principal coordinates analysis (PCoA) based on Bray–Curtis distances to assess β-diversity. PCoA revealed clear separation between the treatment groups (acupuncture and estazolam) and the model group, suggesting significant alterations in gut microbial structure post-intervention. Each point represents an individual sample.

β-Diversity. Principal coordinates analysis (PCoA) based on Bray–Curtis distances revealed clear separation of microbial community structures between the model group and both treatment groups, demonstrating that acupuncture and estazolam each significantly alter gut microecology (Figure 9).

**FIGURE 10:**
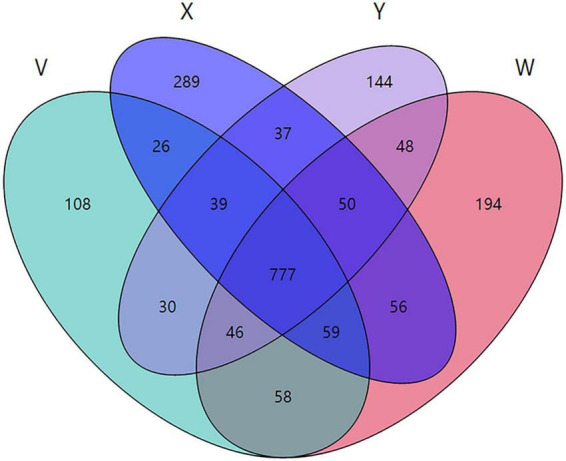
Venn diagram of shared and unique species among the four groups. A total of 777 species were shared across groups. The blank group had 194 unique species, the model group 289, the acupuncture group 144, and the estazolam group 108, reflecting group-specific microbial signatures.

Species-level Venn Analysis. A total of 777 species were shared across all four groups. Unique species counts were 194 in the control group, 289 in the model group, 144 in the acupuncture group, and 108 in the estazolam group, indicating differential maintenance of specific taxa by each intervention (Figure 10).

**FIGURE 11:**
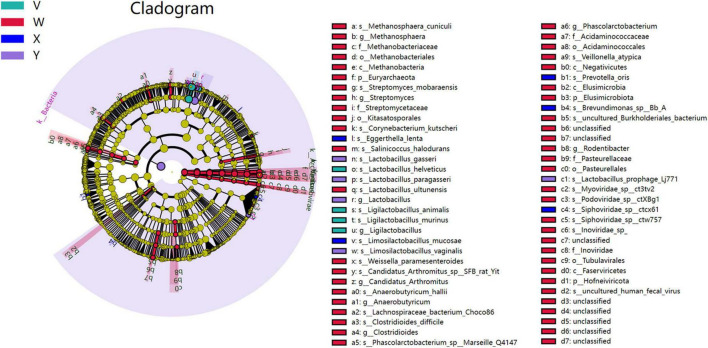
Cladogram based on LEfSe analysis showing significantly enriched taxa across groups. Colors indicate taxa significantly enriched in each group, and node size corresponds to relative abundance. Bacteroidota-related taxa were enriched in the blank group, Eggerthella-related taxa in the model group, and Lactobacillus and its derivatives in both the acupuncture and estazolam groups.

**FIGURE 12:**
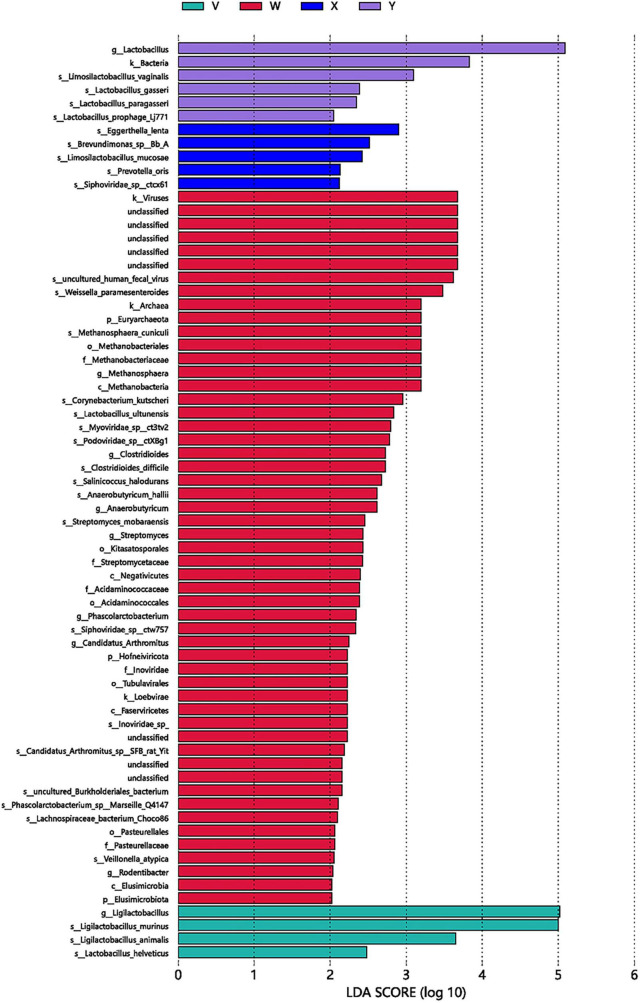
LEfSe LDA score plot of significantly enriched taxa among groups (LDA > 2, *P* < 0.05). A total of 50, 5, 5, and 4 taxa were significantly enriched in the blank, model, acupuncture, and estazolam groups, respectively. Higher LDA scores indicate greater representation of the taxa in the corresponding group. W, blank group; X, model group; Y, acupuncture group; V, estazolam group.

LEfSe Analysis. Differentially enriched taxa (LDA > 2, *P* < 0.05) numbered 50 in the control group, 5 in the model group, 5 in the acupuncture group, and 4 in the estazolam group (Figures 11, 12). In the model group, the most enriched lineage was Actinomycetota (class Coriobacteriia, order Eggerthellales, family Eggerthellaceae, genus Eggerthella, species E. lenta). The acupuncture group showed enrichment of Bacillota (class Bacilli, order Lactobacillales, family Lactobacillaceae, genus Lactobacillus; LDA > 4), whereas the estazolam group was characterized by enrichment of Ligilactobacillus (family Lactobacillaceae; LDA > 4), suggesting that distinct microbial mechanisms underlie the effects of each treatment.

#### 3.3.4 Gut microbiota might influence responses to AP by mediating metabolic pathways

Various metagenomic analyses were performed to explore functional differences in the gut microbiome under different interventions and their potential mechanisms.

**FIGURE 13:**
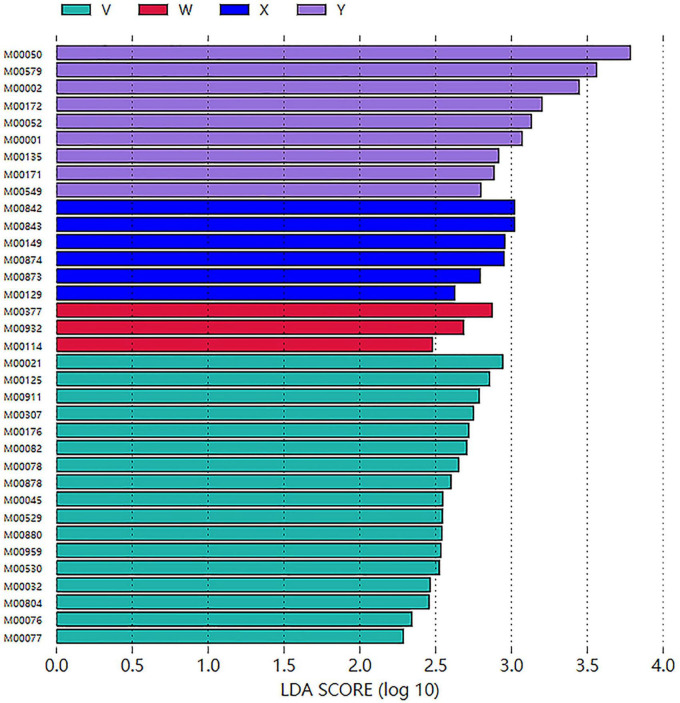
LEfSe analysis of significantly enriched KEGG Orthologs (KOs) among groups (LDA > 2, *P* < 0.05). The plot illustrates the number and LDA scores of functional KOs significantly enriched in each group, indicating differential effects of interventions on microbial functional profiles.

KEGG Functional Annotation. Metagenomic reads were annotated against the KEGG database, and LEfSe analysis was conducted based on LDA > 2 (P < 0.05). Enrichment counts of KEGG Orthologs (KOs) were 3 in the control group, 6 in the model group, 9 in the acupuncture group, and 17 in the estazolam group, indicating distinct functional gene compositions among the groups (Figure 13).

**FIGURE 14:**
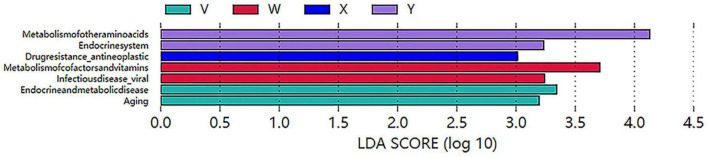
LDA analysis of KEGG level 2 functional pathways significantly enriched in different groups (LDA > 2, *P* < 0.05). The blank group was mainly enriched in cofactor and vitamin metabolism, the model group in drug resistance pathways, the acupuncture group in amino acid metabolism, and the estazolam group in endocrine and metabolic disease-related pathways.

Secondary Pathway Enrichment. At level2, the control group was enriched in metabolism of cofactors and vitamins; the model group in drug resistance–antineoplastic pathways; the acupuncture group in metabolism of other amino acids; and the estazolam group in endocrine and metabolic disease pathways. These patterns suggest that acupuncture and estazolam modulate host metabolism via different mechanisms (Figure 14).

**FIGURE 15:**
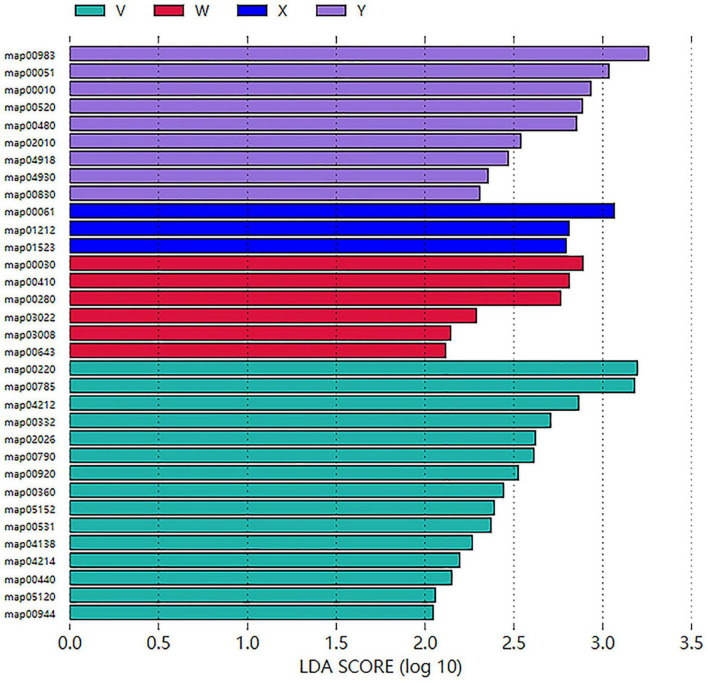
LDA analysis of KEGG level 3 pathways significantly enriched in each group (LDA > 2, *P* < 0.05). Representative KEGG pathways enriched in each group are displayed, reflecting microbial functional differentiation in energy metabolism, carbon utilization, and nutrient biosynthesis.

Key Pathway Identification and Visualization. Subsequent LEfSe LDA analysis identified group-specific enriched pathways (Figure 15). Finally, the Glycolysis/Gluconeogenesis pathway was visualized with KO-level significance coloring, highlighting enzyme-encoding genes significantly enriched in both the acupuncture and control groups. This visualization reveals differential regulation of energy metabolism among interventions (Supplementary Figure S3).

## 4 Discussion

In this PCPA-induced insomnia model, it was observed that acupuncture at the Back-Shu acupoints significantly improved sleep behavior. Compared with the model group, the acupuncture group exhibited a shorter sleep latency (Supplementary Figure S1), consistent with previous reports that acupuncture can improve physiological indicators of insomnia (Yu et al., 2022).

Acupuncture also regulated gut microbiota diversity: PCPA treatment reduced α-diversity, which was partially restored by acupuncture. β-diversity analyses demonstrated that the post-treatment microbiota in the acupuncture group more closely resembled that of the control group rather than the untreated model group. This aligns with Hong’s (Hong et al., 2020) findings that acupuncture shifts the gut microbiota toward a healthy profile, whereas both the insomnia model and zolpidem groups remain dysbiotic.

We further observed significant alterations in key genera. Acupuncture increased the relative abundance of beneficial bacteria such as Lactobacillus, which was markedly depleted in PCPA-treated rats, and suppressed certain overgrown genera associated with insomnia (Figure 3), indicating partial restoration of gut homeostasis. Yu et al. reported that warm acupuncture reverses PCPA-induced declines in Lactobacillus, alleviating dysbiosis (Yu et al., 2022). Metagenomic sequencing in our study identified Lactobacillus johnsonii and Ligilactobacillus murinus as significantly enriched in the acupuncture group (Figure 5), whereas their levels remained low in the estazolam group.

We findings that the therapeutic efficacy of acupuncture for insomnia is accompanied by pronounced reconstruction of the gut microbiota, echoing the growing evidence linking sleep regulation to microbial composition (Huangfu et al., 2019; Ao Guo and Wenbin, 2025). Although both acupuncture and estazolam improved insomnia symptoms, their effects on microbial communities differed. Prior studies indicate that acupuncture can match or surpass pharmacological treatments in enriching beneficial phyla such as Lactobacillus (Zhou et al., 2022). Our results confirm that the acupuncture group exhibited greater probiotic enrichment and upregulation of health-related microbial functions than the estazolam group. In summary, improvements in sleep behavior and gut microbiota rebalancing validate acupuncture’s efficacy and highlight its unique mechanism in modulating gut microecology.

### 4.1 Acupuncture’s potential mechanism: the gut microbiota-gut-brain axis

Acupuncture may primarily act on insomnia via the complex microbiota-gut-brain axis. In this study, acupuncture markedly enriched various Lactobacillus spp., including Lactobacillus johnsonii and Ligilactobacillus murinus, both known to produce neuroactive metabolites and maintain host homeostasis. Previous research has demonstrated that many Lactobacillus strains function as probiotics with sleep-promoting potential (Bongiovanni et al., 2025). These bacteria ferment dietary fiber into short-chain fatty acids (SCFAs) such as butyrate and acetate (Braniste et al., 2014) and convert glutamate into the inhibitory neurotransmitter GABA (Bravo et al., 2011).Such metabolites can directly influence the nervous system and indirectly modulate it via immune pathways and the vagus nerve (Kowalski and Mulak, 2019; Zhu et al., 2020). For example, increased butyrate production correlates with upregulation of host clock genes and enhanced sleep efficiency (Firoozi et al., 2024), while butyrate also strengthens the intestinal barrier and exerts anti-inflammatory and neuroregulatory effects, including microglial modulation (Hodgkinson et al., 2023). Although exogenous GABA poorly crosses the blood–brain barrier, microbe-derived GABA may affect central GABAergic activity via gut mucosal neurons, the vagus nerve, or peripheral immune routes (Yarandi et al., 2016). Animal studies show that oral L. johnsonii reduces brain glutamate and increases GABA in stressed mice, alleviating anxiety and improving cognition (Wang et al., 2020), consistent with findings that specific lactic acid bacteria alter brain GABA receptor expression to reduce anxiety- and depression-like behaviors (Bravo et al., 2011). In our study, the significant post-acupuncture increase in L. johnsonii may promote GABA and other sleep-enhancing metabolites, thereby improving sleep.

Moreover, both L. johnsonii and L. murinus regulate immunity and gut barrier integrity. L. johnsonii upregulates tight-junction proteins, enhancing epithelial integrity and modulating immunity (Arzola-Martínez et al., 2024). In rodents, L. murinus supplementation increases claudin-4 expression, reduces intestinal permeability, circulating endotoxin, and TNF-α levels (Mukohda et al., 2023). These findings suggest that acupuncture-induced enrichment of L. murinus may protect against PCPA-nduced barrier dysfunction, mitigating systemic inflammation and HPA axis overactivation—processes integral to insomnia. Additionally, dominance of SCFA-producing Gram-positive bacteria can establish a positive feedback loop: a robust gut barrier and low chronic inflammation alleviate insomnia-associated neurochemical imbalances (Hodgkinson et al., 2023), synergizing with acupuncture’s neuromodulatory effects to enhance sleep.

Acupuncture altered pathways of amino acid and carbohydrate metabolism. Tryptophan, a precursor of serotonin and melatonin, is pivotal for sleep regulation (Zhang et al., 2022). Upregulation of microbial tryptophan metabolism may influence host serotonin or produce bioactive derivatives, partly explaining acupuncture’s tendency to restore 5-HT and melatonin in insomnia models (Fang et al., 2024). Changes in glycolysis and energy metabolism pathways likely reflect increased microbial activity and metabolite production (van der Hee and Wells, 2021). SCFAs, as fermentation end-products, supply host energy and modulate immune cells and enteric neurons via G-protein–coupled receptors (Kim, 2023). SCFAs also mitigate stress-induced HPA axis overactivation and reduce gut permeability, counteracting insomnia pathophysiology (Quagebeur et al., 2023). Collectively, acupuncture-driven enhancements in SCFA and neurotransmitter synthesis underscore its roles in improving sleep and reducing inflammation. By modulating the microbiota, acupuncture may activate the PI3K/Akt pathway, promoting cell survival and anti-inflammatory effects peripherally and centrally, supporting neurogenesis and suppressing excessive inflammation to improve sleep and mood (Krawczyk et al., 2025).

Although PI3K/Akt activity was not directly measured, we hypothesize that SCFA enrichment—particularly butyrate—induces gut-derived gluconeogenesis (Ji et al., 2021) and may activate PI3K/Akt in enteric glia and immune cells (Wang H. et al., 2021). Enhanced gut-brain interactions could then elevate BDNF and GABA in the brain (Marx et al., 2021). In summary, acupuncture’s effect in insomnia represents a holistic rebalancing of the gut–brain axis: reshaping the microbiota while generating beneficial metabolic and immune signals that, alongside direct neuromodulation, collaboratively alleviate sleep disturbances (Eshkevari et al., 2013; Elieh Ali Komi et al., 2020; Wang et al., 2023). These findings align with prior evidence that correcting dysbiosis alleviates insomnia and anxiety by modulating sleep-related neurotransmitters and neuroinflammation.

### 4.2 Model validity and rationale for acupoint selection

In this study, insomnia was induced in rats by intraperitoneal injection of para-chlorophenylalanine (PCPA), a well-validated model based on its biochemical mechanism (Bao et al., 2024; Fu et al., 2024; Wang et al., 2024). Model establishment was confirmed by the disappearance of the righting reflex following PCPA administration (Tian et al., 2024). A One-week observation period was adopted based on previous preclinical and clinical reports (Qianna Wang, 2022; Jiahui et al., 2024; Wei Li et al., 2024). In our experiment, acupuncture was applied for 7 days starting 24 hours after the final PCPA injection. This timeframe aligns with the commonly accepted validity window of the PCPA model and avoids overlap with the spontaneous recovery phase typically observed after day 9 (Tian et al., 2024). Thus, the therapeutic effects observed in this study are more likely attributable to the intervention rather than natural remission. Future research may include longer observation periods or a delayed-treatment design to examine acupuncture’s role in the recovery phase.

Acupoints were selected according to classical Chinese medicine theory, targeting the Back-Shu points of the five Zang organs: Heart Shu (BL15), Spleen Shu (BL20), and Kidney Shu (BL23). These points are traditionally used to regulate organ function and strengthen vital qi, and are commonly employed in clinical treatment of sleep disorders (Qiyun Liu, 2021; Aiyu Wang, 2023). Previous clinical studies have reported that needling these Back-Shu points significantly reduces Pittsburgh Sleep Quality Index scores, with efficacy rates up to 95.5% (Qianna Wang, 2022). As this study did not include a direct comparison with other acupoint combinations, definitive conclusions regarding relative superiority cannot be drawn. Nevertheless, our results support the use of Back-Shu points as a classical and effective acupoint selection strategy for insomnia management in traditional Chinese medicine.

### 4.3 Limitations of the study

This study has several limitations. First, the findings are mainly correlational, and it remains unclear whether the observed microbiota changes directly contribute to the improvements in sleep. Future studies using fecal microbiota transplantation or antibiotic depletion are needed to determine causality. Second, although metagenomic predictions suggested involvement of pathways such as SCFA synthesis and amino acid metabolism, we did not directly measure related metabolites or host signaling molecules. Validation through biochemical and molecular assays is needed. Third, the sample size was relatively small, which may have limited the ability to detect subtle differences, particularly given the variability in gut microbiota among individuals. Fourth, only male rats were used. As sex hormones can influence gut–brain interactions and sleep physiology, future studies should include both sexes to assess sex-specific responses.

Finally, behavioral assessments were limited. Descriptions of improved mental state, increased activity, and reduced depressive-like features were based on sleep latency tests and daily observations. Standardized behavioral tests were not performed, and the open-field test was brief. The rotarod test may also be affected by motor coordination rather than sedation. Future work should include more objective and comprehensive behavioral measures to strengthen these findings.

While this study focused on the acute stage of PCPA-induced insomnia, and interventions were limited to the first 7 days, future studies could incorporate extended timepoints or include delayed interventions to determine whether acupuncture has a role in modulating the spontaneous recovery phase.

## 5 Conclusion

These findings indicate that acupuncture at Back-Shu points not only alleviates insomnia-like behaviors in rats but also remodels the gut microbiota and its associated metabolic pathways, offering preliminary support for the “microecology–gut–brain axis” as a mechanistic basis for acupuncture-based insomnia therapy. By bridging traditional Chinese medicine and modern biomedical research, this work lays a theoretical foundation for a novel, gut-centered treatment paradigm for insomnia. Confirmation of similar microecological changes correlated with sleep improvements in clinical populations would support the development of more personalized and integrative insomnia therapies.

## Data Availability

The data presented in this study are deposited in the Zenodo repository, publicly available at https://zenodo.org/records/15567717.

## Supplementary material

**Supplementary Figure S1** Bar graph of sleep latency and sleep duration among groups after modeling. Data are presented as mean ± standard deviation; *P* < 0.05, *P* < 0.01, *P* < 0.001; ns indicates no statistically significant difference.

**Supplementary Figure S2** Bar graph of sleep latency and sleep duration among groups after treatment. Data are presented as mean ± standard deviation; *P* < 0.05, *P* < 0.01, *P* < 0.001; ns indicates no statistically significant difference.

**Supplementary Figure S3** Functional visualization map of the glycolysis/gluconeogenesis pathway. Notes: The diagram shows the positions of metabolic enzymes corresponding to KEGG Orthologs (KOs) significantly enriched in each group. Red nodes represent metabolic sites enriched in the blank group, while purple nodes indicate key gene loci enriched in the acupuncture group, suggesting differential regulation in energy metabolism pathways.

## References

[B1] Aiyu Wang Q. (2023). Effects of balanced cupping combined with five shu points massage on symptoms, inflammatory markers, and sleep quality in children with cough due to phlegm-dampness accumulating in the lung syndrome. *J. World Sleep Med.* 10 1762–1764. 10.3969/j.issn.2095-7130.2023.08.011

[B2] Ao Guo D. Wenbin M. (2025). Characterization of gut microbiota in mice receiving fecal microbiota transplantation from chronic insomnia patients. *J. Binzhou Med. Univ.* 48 7–11. 10.19739/j.cnki.issn1001-9510.2025.01.002

[B3] Arzola-Martínez L. Ravi K. Huffnagle G. Lukacs N. Fonseca W. (2024). *Lactobacillus johnsonii* and host communication: Insight into modulatory mechanisms during health and disease. *Front. Microbiomes* 2:1345330. 10.3389/frmbi.2023.1345330 PMC12993539 41853382

[B4] Bao Y. Zhou H. Fu Y. Wang C. Huang Q. (2024). Zhumian Granules improves PCPA-induced insomnia by regulating the expression level of neurotransmitters and reducing neuronal apoptosis. *J. Ethnopharmacol.* 327:118048. 10.1016/j.jep.2024.118048 38484955

[B5] Bongiovanni T. Santiago M. Zielinska K. Scheiman J. Barsa C. Jäger R. (2025). A Lactobacillus consortium provides insights into the sleep-exercise-microbiome nexus in proof of concept studies of elite athletes and in the general population. *Microbiome* 13:1. 10.1186/s40168-024-01936-4 39748236 PMC11697739

[B6] Braniste V. Al-Asmakh M. Kowal C. Anuar F. Abbaspour A. Tóth M. (2014). The gut microbiota influences blood-brain barrier permeability in mice. *Sci. Transl. Med.* 6:263ra158. 10.1126/scitranslmed.3009759 25411471 PMC4396848

[B7] Bravo J. Forsythe P. Chew M. Escaravage E. Savignac H. Dinan T. (2011). Ingestion of Lactobacillus strain regulates emotional behavior and central GABA receptor expression in a mouse via the vagus nerve. *Proc. Natl. Acad. Sci. U. S. A.* 108 16050–16055. 10.1073/pnas.1102999108 21876150 PMC3179073

[B8] Brum J. Ignacio-Espinoza J. Roux S. Doulcier G. Acinas S. Alberti A. (2015). Ocean plankton. Patterns and ecological drivers of ocean viral communities. *Science* 348:1261498. 10.1126/science.1261498 25999515

[B9] Buchfink B. Xie C. Huson D. (2015). Fast and sensitive protein alignment using DIAMOND. *Nat. Methods* 12 59–60. 10.1038/nmeth.3176 25402007

[B10] Elieh Ali Komi D. Wöhrl S. Bielory L. (2020). Mast cell biology at molecular level: A comprehensive review. *Clin. Rev. Allergy Immunol.* 58 342–365. 10.1007/s12016-019-08769-2 31828527

[B11] Eshkevari L. Permaul E. Mulroney S. (2013). Acupuncture blocks cold stress-induced increases in the hypothalamus-pituitary-adrenal axis in the rat. *J. Endocrinol.* 217 95–104. 10.1530/JOE-12-0404 23386059

[B12] Fang J. Wang S. Liu L. Zhang X. Liu R. Pang X. (2024). Gut microbiota: A potential influencer of insomnia occurring after COVID-19 infection. *Front. Psychiatry* 15:1423715. 10.3389/fpsyt.2024.1423715 39109368 PMC11300359

[B13] Firoozi D. Masoumi S. Mohammad-Kazem Hosseini Asl S. Labbe A. Razeghian-Jahromi I. Fararouei M. (2024). Effects of short-chain fatty acid-butyrate supplementation on expression of circadian-clock genes, sleep quality, and inflammation in patients with active ulcerative colitis: A double-blind randomized controlled trial. *Lipids Health Dis.* 23:216. 10.1186/s12944-024-02203-z 39003477 PMC11245831

[B14] Ford D. Kamerow D. (1989). Epidemiologic study of sleep disturbances and psychiatric disorders. An opportunity for prevention? *JAMA* 262 1479–1484. 10.1001/jama.262.11.1479 2769898

[B15] Franzosa E. McIver L. Rahnavard G. Thompson L. Schirmer M. Weingart G. (2018). Species-level functional profiling of metagenomes and metatranscriptomes. *Nat. Methods* 15 962–968. 10.1038/s41592-018-0176-y 30377376 PMC6235447

[B16] Fu X. Yan S. Hu Z. Sheng W. Li W. Kuang S. (2024). Guhan Yangsheng Jing mitigates hippocampal neuronal pyroptotic injury and manifies learning and memory capabilities in sleep deprived mice via the NLRP3/Caspase1/GSDMD signaling pathway. *J. Ethnopharmacol.* 326:117972. 10.1016/j.jep.2024.117972 38403005

[B17] Hodgkinson K. El Abbar F. Dobranowski P. Manoogian J. Butcher J. Figeys D. (2023). Butyrate’s role in human health and the current progress towards its clinical application to treat gastrointestinal disease. *Clin. Nutr.* 42 61–75. 10.1016/j.clnu.2022.10.024 36502573

[B18] Hong J. Chen J. Kan J. Liu M. Yang D. (2020). Effects of acupuncture treatment in reducing sleep disorder and gut microbiota alterations in PCPA-induced insomnia mice. *Evid. Based Complement. Alternat. Med.* 2020:3626120. 10.1155/2020/3626120 33178314 PMC7647758

[B19] Huangfu Y. Peng W. Guo B. Shen Z. Li L. Liu S. (2019). Effects of acupuncture in treating insomnia due to spleen-stomach disharmony syndrome and its influence on intestinal microbiome: Study protocol for a randomized controlled trial. *J. Integr. Med.* 17 161–166. 10.1016/j.joim.2019.01.007 30819614

[B20] Ji J. Sun Q. Nie D. Wang Q. Zhang H. Qin F. (2021). Probiotics protect against RSV infection by modulating the microbiota-alveolar-macrophage axis. *Acta Pharmacol. Sin.* 42 1630–1641. 10.1038/s41401-020-00573-5 33495515 PMC8463687

[B21] Jiahui Q. Xiyan G. Shangwen Q. Yubing L. Yujie L. Wei L. (2024). Effects of acupuncture at back-shu points on hypothalamic STAT3, Th17/Treg balance, and related inflammatory cytokine levels in insomnia model rats. *J. Clin. Acupunct. Moxibust.* 40 72–78. 10.19917/j.cnki.1005-0779.024237

[B22] Kechin A. Boyarskikh U. Kel A. Filipenko M. (2017). cutPrimers: A new tool for accurate cutting of primers from reads of targeted next generation sequencing. *J. Comput. Biol.* 24 1138–1143. 10.1089/cmb.2017.0096 28715235

[B23] Kim C. (2023). Complex regulatory effects of gut microbial short-chain fatty acids on immune tolerance and autoimmunity. *Cell Mol. Immunol.* 20 341–350. 10.1038/s41423-023-00987-1 36854801 PMC10066346

[B24] Kim J. Kim M. Koh A. Xie Y. Zhan X. (2016). FMAP: Functional mapping and analysis pipeline for metagenomics and metatranscriptomics studies. *BMC Bioinformatics* 17:420. 10.1186/s12859-016-1278-0 27724866 PMC5057277

[B25] Kowalski K. Mulak A. (2019). Brain-gut-microbiota axis in Alzheimer’s Disease. *J. Neurogastroenterol. Motil.* 25 48–60. 10.5056/jnm18087 30646475 PMC6326209

[B26] Krawczyk A. Sladowska G. Strzalka-Mrozik B. (2025). The role of the gut microbiota in modulating signaling pathways and oxidative stress in glioma therapies. *Cancers* 17:719. 10.3390/cancers17050719 40075568 PMC11899293

[B27] Langmead B. Salzberg S. (2012). Fast gapped-read alignment with Bowtie 2. *Nat. Methods* 9 357–359. 10.1038/nmeth.1923 22388286 PMC3322381

[B28] Lee B. Kim B. Kim M. Kim A. Park H. Kwon O. (2022). Electroacupuncture for treating cancer-related insomnia: A multicenter, assessor-blinded, randomized controlled, pilot clinical trial. *BMC Complement. Med. Ther.* 22:77. 10.1186/s12906-022-03561-w 35303841 PMC8932204

[B29] Lin Z. Jiang T. Chen M. Ji X. Wang Y. (2024). Gut microbiota and sleep: Interaction mechanisms and therapeutic prospects. *Open Life Sci.* 19:20220910. 10.1515/biol-2022-0910 39035457 PMC11260001

[B30] Lu J. Breitwieser F. Thielen P. Salzberg S. (2017). Bracken: Estimating species abundance in metagenomics data. *PeerJ Comput. Sci.* 3:e104. 10.7717/peerj-cs.104 40271438 PMC12016282

[B31] Madari S. Golebiowski R. Mansukhani M. Kolla B. (2021). Pharmacological management of insomnia. *Neurotherapeutics* 18 44–52. 10.1007/s13311-021-01010-z 33527255 PMC8116439

[B32] Mandal S. Van Treuren W. White R. Eggesbø M. Knight R. Peddada S. (2015). Analysis of composition of microbiomes: A novel method for studying microbial composition. *Microb. Ecol. Health Dis.* 26:27663. 10.3402/mehd.v26.27663 26028277 PMC4450248

[B33] Martin M. (2011). Cutadapt removes adapter sequences from high-throughput sequencing reads. *EMBnetjournal* 17 10–12. 10.14806/ej.17.1.200

[B34] Marx W. Lane M. Hockey M. Aslam H. Berk M. Walder K. (2021). Diet and depression: Exploring the biological mechanisms of action. *Mol. Psychiatry* 26 134–150. 10.1038/s41380-020-00925-x 33144709

[B35] Morin C. Jarrin D. Ivers H. Mérette C. LeBlanc M. Savard J. (2020). Incidence, persistence, and remission rates of insomnia over 5 years. *JAMA Netw. Open* 3:e2018782. 10.1001/jamanetworkopen.2020.18782 33156345 PMC7648256

[B36] Morphy H. Dunn K. Lewis M. Boardman H. Croft P. (2007). Epidemiology of insomnia: A longitudinal study in a UK population. *Sleep* 30 274–280. 10.1093/sleep/30.3.274 17425223

[B37] Mukohda M. Yano T. Matsui T. Nakamura S. Miyamae J. Toyama K. (2023). Treatment with *Ligilactobacillus murinus* lowers blood pressure and intestinal permeability in spontaneously hypertensive rats. *Sci. Rep.* 13:15197. 10.1038/s41598-023-42377-7 37709803 PMC10502128

[B38] Pan L. Hong Z. Guan R. (2024). Research progress on insomnia treated by traditional Chinese medicine and acupuncture based on microbial-gut-brain axis theory. *World J. Clin. Cases* 12 3314–3320. 10.12998/wjcc.v12.i18.3314 38983433 PMC11229893

[B39] Perlis M. Posner D. Riemann D. Bastien C. Teel J. Thase M. (2022). Insomnia. *Lancet* 400 1047–1060. 10.1016/S0140-6736(22)00879-0 36115372

[B40] Pillai V. Roth T. Drake C. (2015). The nature of stable insomnia phenotypes. *Sleep* 38 127–138. 10.5665/sleep.4338 25325468 PMC4262945

[B41] Pillai V. Roth T. Drake C. (2016). Towards quantitative cutoffs for insomnia: How current diagnostic criteria mischaracterize remission. *Sleep Med.* 26 62–68. 10.1016/j.sleep.2016.01.013 27288048 PMC4983260

[B42] Qianna Wang L. (2022). Clinical observation on 66 cases of refractory insomnia treated with Yuan-Luo-Back-Shu point combination method. *J. Chin. Acupunct.* 42 369–370. 10.13703/j.0255-2930.20210302-k0001 30942026

[B43] Qiyun Liu Z. (2021). Clinical observation on acupuncture treatment of back-shu points for geriatric insomnia with heart-spleen deficiency syndrome. *J. Chin. Gerontol.* 41 5024–5027. 10.3969/j.issn.1005-9202.2021.22.046

[B44] Quagebeur R. Dalile B. Raes J. Van Oudenhove L. Verbeke K. Vrieze E. (2023). The role of short-chain fatty acids (SCFAs) in regulating stress responses, eating behavior, and nutritional state in anorexia nervosa: Protocol for a randomized controlled trial. *J. Eat. Disord.* 11:191. 10.1186/s40337-023-00917-6 37884972 PMC10605799

[B45] Schmieder R. Edwards R. (2011). Quality control and preprocessing of metagenomic datasets. *Bioinformatics* 27 863–864. 10.1093/bioinformatics/btr026 21278185 PMC3051327

[B46] Segata N. Izard J. Waldron L. Gevers D. Miropolsky L. Garrett W. (2011). Metagenomic biomarker discovery and explanation. *Genome Biol.* 12:R60. 10.1186/gb-2011-12-6-r60 21702898 PMC3218848

[B47] Sejbuk M. Siebieszuk A. Witkowska A. (2024). The role of gut microbiome in sleep quality and health: Dietary strategies for microbiota support. *Nutrients* 16:2259. 10.3390/nu16142259 39064702 PMC11279861

[B48] Sweetman A. Putland S. Lack L. McEvoy R. Adams R. Grunstein R. (2021). The effect of cognitive behavioural therapy for insomnia on sedative-hypnotic use: A narrative review. *Sleep Med. Rev.* 56:101404. 10.1016/j.smrv.2020.101404 33370637

[B49] Tian T. Meng Z. Yuan W. Lian L. Xuehui S. (2024). Exploration of the effect of electroacupuncture on MFG-E8 in PCPA-induced insomnia rats based on the pivot theory. *J. Tradit. Chin. Med. Mater. Med.* 45 69–74. 10.16254/j.cnki.53-1120/r.2024.11.016

[B50] van der Hee B. Wells J. (2021). Microbial regulation of host physiology by short-chain fatty acids. *Trends Microbiol.* 29 700–712. 10.1016/j.tim.2021.02.001 33674141

[B51] Villar E. Farrant G. Follows M. Garczarek L. Speich S. Audic S. (2015). Ocean plankton. Environmental characteristics of Agulhas rings affect interocean plankton transport. *Science* 348:1261447. 10.1126/science.1261447 25999514

[B52] Wang C. Xu W. Li G. Fu C. Li J. Wang J. (2021). Impact of acupuncture on sleep and comorbid symptoms for chronic insomnia: A randomized clinical trial. *Nat. Sci. Sleep* 13 1807–1822. 10.2147/NSS.S326762 34675728 PMC8519353

[B53] Wang H. Liu F. Li R. Wan M. Li J. Shi J. (2021). Electroacupuncture improves learning and memory functions in a rat cerebral ischemia/reperfusion injury model through PI3K/Akt signaling pathway activation. *Neural Regen. Res.* 16 1011–1016. 10.4103/1673-5374.300454 33269744 PMC8224106

[B54] Wang H. Sun Y. Xin J. Zhang T. Sun N. Ni X. (2020). *Lactobacillus johnsonii* BS15 prevents psychological stress-induced memory dysfunction in mice by modulating the gut-brain axis. *Front. Microbiol.* 11:1941. 10.3389/fmicb.2020.01941 32903531 PMC7438410

[B55] Wang M. Liu W. Ge J. Liu S. (2023). The immunomodulatory mechanisms for acupuncture practice. *Front. Immunol.* 14:1147718. 10.3389/fimmu.2023.1147718 37090714 PMC10117649

[B56] Wang Z. Li D. Chen M. Yu X. Chen C. Chen Y. (2024). A comprehensive study on the regulation of Compound Zaoren Granules on cAMP/CREB signaling pathway and metabolic disorder in CUMS-PCPA induced insomnia rats. *J. Ethnopharmacol.* 332:118401. 10.1016/j.jep.2024.118401 38815875

[B57] Wei L. Congying L. Xiyan G. Yujie L. Yubing L. Jiahui Q. (2024). Effect of Acupuncture with Yuan-Shu point combination on the p38MAPK signaling pathway in the brain tissue of insomnia-induced rats. *J. Shaanxi Tradit Chin. Med.* 45 883–887. 10.3969/j.issn.1000-7369.2024.07.004

[B58] Wong M. Brower K. Craun E. (2016). Insomnia symptoms and suicidality in the National Comorbidity Survey - Adolescent Supplement. *J. Psychiatr. Res.* 81 1–8. 10.1016/j.jpsychires.2016.06.004 27355426 PMC5021568

[B59] Wood D. Salzberg S. (2014). Kraken: Ultrafast metagenomic sequence classification using exact alignments. *Genome Biol.* 15:R46. 10.1186/gb-2014-15-3-r46 24580807 PMC4053813

[B60] Xu D. Zhao S. Cui J. Ma T. Xu B. Yu X. (2019). [A new attempt of re-mapping acupoint atlas in the rat]. *Zhen Ci Yan Jiu* 44 62–65. 10.13702/j.1000-0607.180396 30773865

[B61] Yarandi S. Peterson D. Treisman G. Moran T. Pasricha P. (2016). Modulatory effects of gut microbiota on the central nervous system: How gut could play a role in neuropsychiatric health and diseases. *J. Neurogastroenterol. Motil.* 22 201–212. 10.5056/jnm15146 27032544 PMC4819858

[B62] Yeung W. Yu B. Yuen J. Ho J. Chung K. Zhang Z. (2021). Semi-individualized acupuncture for insomnia disorder and oxidative stress: A randomized, double-blind, sham-controlled trial. *Nat. Sci. Sleep* 13 1195–1207. 10.2147/NSS.S318874 34321944 PMC8310926

[B63] Yu H. Yu H. Si L. Meng H. Chen W. Wang Z. (2022). Influence of warm acupuncture on gut microbiota and metabolites in rats with insomnia induced by PCPA. *PLoS One* 17:e0267843. 10.1371/journal.pone.0267843 35482778 PMC9049555

[B64] Zhang Y. Lang R. Guo S. Luo X. Li H. Liu C. (2022). Intestinal microbiota and melatonin in the treatment of secondary injury and complications after spinal cord injury. *Front. Neurosci.* 16:981772. 10.3389/fnins.2022.981772 36440294 PMC9682189

[B65] Zhao F. Spencer S. Kennedy G. Zheng Z. Conduit R. Zhang W. (2024). Acupuncture for primary insomnia: Effectiveness, safety, mechanisms and recommendations for clinical practice. *Sleep Med. Rev.* 74:101892. 10.1016/j.smrv.2023.101892 38232645

[B66] Zhou Y. Yang S. Wang Y. Lu W. Chen L. Liang F. (2022). [Effect of electroacupuncture at different acupoint combination on intestinal inflammatory response and intestinal flora in obese rats]. *Zhongguo Zhen Jiu* 42 1145–1152. 10.13703/j.0255-2930.20211014-0001 37199206

[B67] Zhu S. Jiang Y. Xu K. Cui M. Ye W. Zhao G. (2020). The progress of gut microbiome research related to brain disorders. *J. Neuroinflamm.* 17:25. 10.1186/s12974-020-1705-z 31952509 PMC6969442

[B68] Zhu W. Lomsadze A. Borodovsky M. (2010). Ab initio gene identification in metagenomic sequences. *Nucleic Acids Res.* 38:e132. 10.1093/nar/gkq275 20403810 PMC2896542

