# Supplementary figures and images for: Effect of acupuncture at Back-Shu points on gut microbiota in insomnia model rats based on metagenomic sequencing technology
Source: Front Microbiol. 2025 Jun 19;16:1541958. doi: 10.3389/fmicb.2025.1541958 (PMC12225647; doi:10.3389/fmicb.2025.1541958)

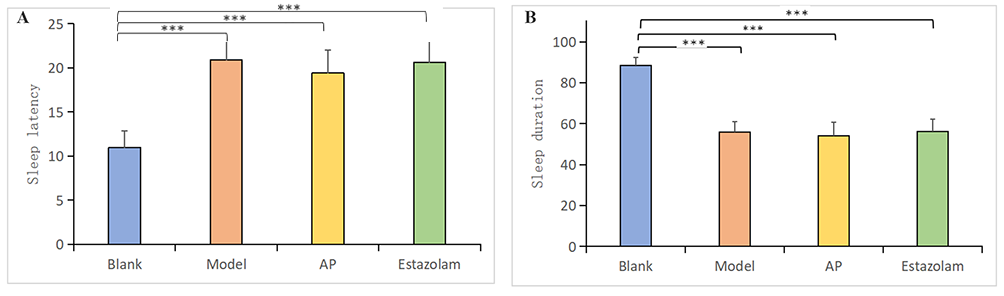

Supplement: Supplementary file 1 [file Image_1.tif]

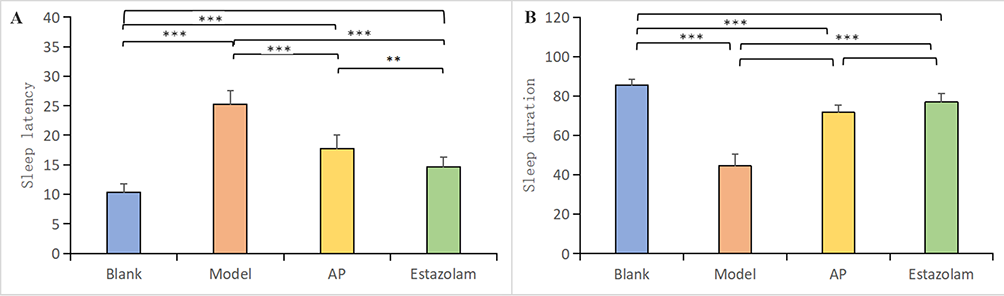

Supplement: Supplementary file 2 [file Image_2.tif]

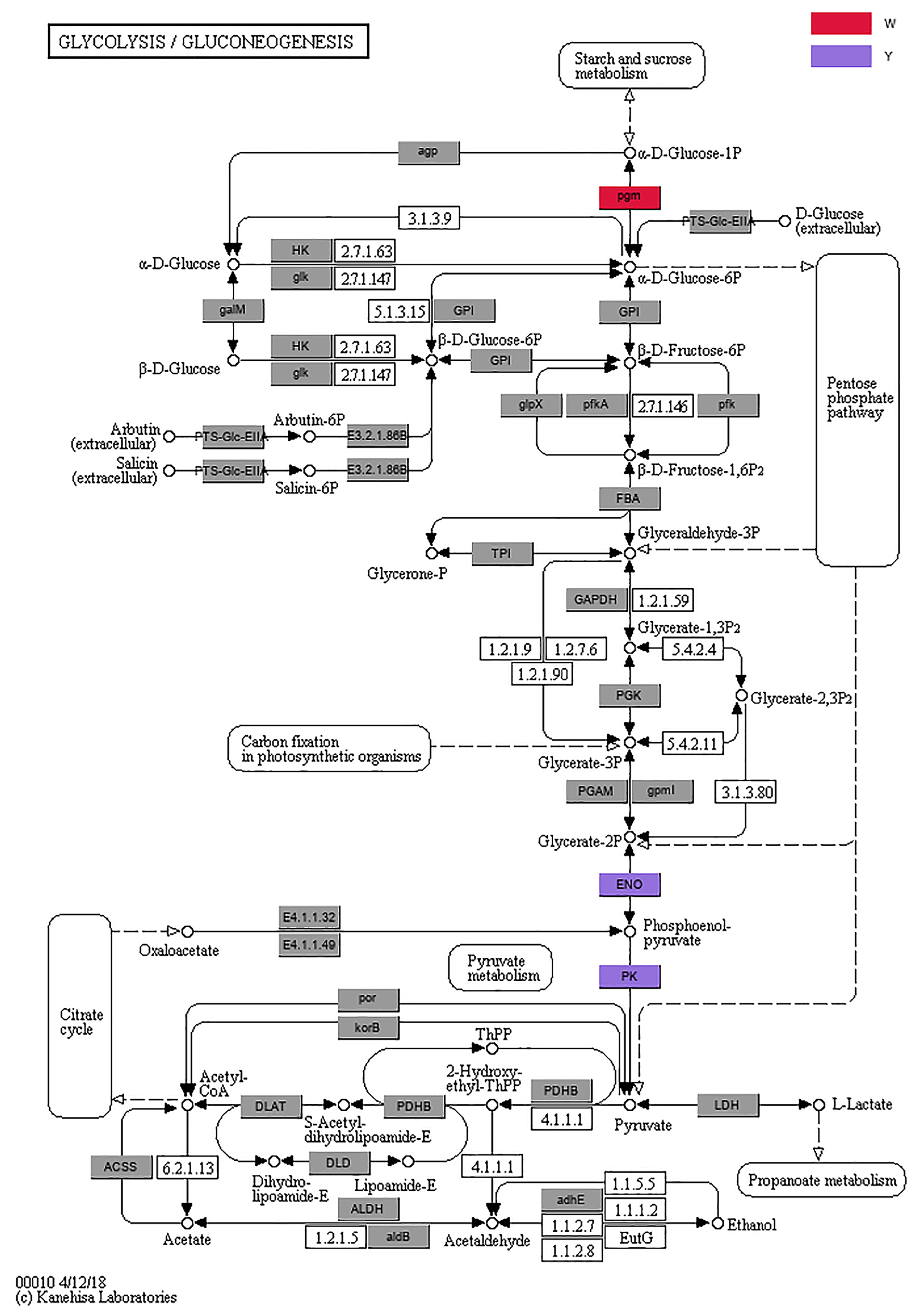

Supplement: Supplementary file 3 [file Image_3.tif]
